# Molecular Epidemiology of *GSTM1* and *GSTT1* Null Genotypes in High-Altitude Andean Populations of Peru

**DOI:** 10.3390/ijms27042009

**Published:** 2026-02-20

**Authors:** Marlon Garcia-Paitan, Carlos Campos-Semino, Zoila Cansinos-Delgado, Milagros Merma-Rosales, Raul Enriquez-Laurente, Saul J. Santivañez, Luis Jaramillo-Valverde

**Affiliations:** 1 School of Medicine, Universidad Continental, Lima 15046, Peru; mgarcia@continental.edu.pe (M.G.-P.); 75491357@continental.edu.pe (C.C.-S.); 71559457@continental.edu.pe (Z.C.-D.); 74850159@continental.edu.pe (M.M.-R.); 2 Research Department, Universidad Continental, Huancayo 12001, Peru; renriquez@continental.edu.pe (R.E.-L.); ssantivanez@continental.edu.pe (S.J.S.)

**Keywords:** *GSTM1*, *GSTT1*, Peru, Andean population

## Abstract

Glutathione-S-Transferase T1 (*GSTT1*) and M1 (*GSTM1*) are key enzymes involved in phase II detoxification. Null genotypes resulting from gene deletions are known to cause a complete loss of enzymatic activity and have been associated with altered xenobiotic metabolism in previous studies. Although genotype frequencies vary across ethnic groups, data from non-European populations, particularly Andean populations, remain limited. In this cross-sectional study, the frequency of *GSTM1* and *GSTT1* null genotypes was determined in 206 individuals from Cusco and Junín. Genotyping was performed by PCR using genomic DNA extracted from peripheral blood. The frequency of the *GSTM1* null genotype was 49.51%, whereas that of *GSTT1* was 25.24%. Combined genotype analysis showed that 63.11% of participants carried at least one null genotype and 11.65% carried both null variants. No significant differences were observed between Cusco and Junín. Compared with previously reported data, these frequencies were similar to those observed in Peruvian coastal and several South American populations. At the intercontinental level, frequencies were comparable to Europe, the Middle East, and Asia but differed from Sub-Saharan Africa and Native American populations. This first molecular characterization of *GSTM1* and *GSTT1* null genotypes in Andean populations provides a population-specific genetic baseline for pharmacogenetics and precision medicine research in high-altitude settings.

## 1. Introduction

Daily exposure to a wide range of xenobiotics, including pharmaceuticals, pesticides, and food additives, necessitates efficient biotransformation processes to prevent clinically relevant adverse effects. The main metabolic detoxification pathways are organized into Phase I (functionalization) and Phase II (conjugation), whose purpose is to increase the hydrophilicity of compounds and reduce their toxicity, thereby facilitating their elimination via bile or urine [1]. During Phase I, the cytochrome P450 enzyme family constitutes the first line of defense against xenobiotics by introducing reactive functional groups such as hydroxyl, carboxyl, or amino groups through oxidation, reduction, or hydrolysis reactions. In Phase II, the metabolites generated are conjugated with hydrophilic molecules through specific enzymes, including glucuronosyltransferases (glucuronic acid), sulfotransferases (sulfate), amino acid transferases, N-acetyltransferases (acetyl group), methyltransferases (methyl group), and glutathione S-transferases (glutathione), thereby increasing their solubility and promoting their excretion. Some xenobiotics may bypass Phase I and be directly metabolized by Phase II enzymes [2], whereas others require only Phase I reactions for their elimination [3].

Polymorphisms in genes encoding xenobiotic-metabolizing enzymes can substantially alter enzymatic activity, with significant implications for chemical clearance, systemic exposure, and internal dose [4]. This topic has been widely explored in pharmacological research, where genetic variability contributes to adverse drug reactions and explains interindividual variability in drug biotransformation and therapeutic outcomes [5]. Among these enzymes, glutathione S-transferases (GSTs), particularly GST Mu 1 (*GSTM1*) and GST theta 1 (*GSTT1*), are of special relevance. Both genes harbor deletion variants (null genotypes) that follow an autosomal recessive pattern and result in complete loss of enzymatic activity only in individuals homozygous for the deletion. These deletions arise from homologous recombination events between flanking repetitive sequences and lead to the complete loss of the corresponding gene. In *GSTM1*, this mechanism produces a deletion of approximately 16 kb encompassing the entire gene (~5.9 kb), whereas in *GSTT1* it generates a deletion of around 54 kb that likewise includes the complete gene (~8.1 kb) [6]. Although other allelic variants of *GSTM1* and *GSTT1*, such as SNPs and small insertions/deletions, have been described, many of these variants have little or no functional impact compared with complete gene deletions [7].

In the literature, these null genotypes have been primarily associated with various cancer types, as well as with therapeutic areas including neuropsychiatry, gastroenterology, respiratory diseases, gynecology, infectious diseases, and cardiology [8]. Additionally, *GSTM1* and *GSTT1* null genotypes have been reported to contribute to variability in the safety and efficacy of drugs such as antiepileptics, immunosuppressants, chemotherapeutic agents, analgesics, and anti-infectives [2,9].

The frequency of *GSTM1* and *GSTT1* null genotypes varies according to ethnicity and continental ancestry, as shown by large-scale population studies and systematic reviews, with differences reported both for individuals carrying a single null genotype and for those carrying combined *GSTM1*/*GSTT1* null genotypes, reflecting population-related differences in clinical susceptibility [8,10]. However, the literature indicates a marked underrepresentation of non-European populations in studies of these genes, resulting in a knowledge gap that restricts progress toward more equitable and inclusive precision medicine [8]. This challenge is further intensified by the possibility that certain variants or rare alleles may be restricted to specific ethnic groups and remain unidentified.

Studies investigating the frequency of *GSTM1* and *GSTT1* null genotypes in populations from the Americas remain limited, accounting for less than 1% of the articles included in a systematic review published in 2022 [8]. In Peru, no previous studies have examined Andean populations, as existing research has been confined to coastal populations [11,12,13,14]. In this context, the aim of the present study was to determine the frequency of *GSTM1* and *GSTT1* null genotypes in Andean populations of Peru. Identifying these genotypes contributes population-specific genetic information that may guide future genetic and molecular epidemiology studies in rural and vulnerable populations.

## 2. Results

The distribution of GST genotype frequencies among Peruvian Andean individuals is summarized in Table 1. In the overall cohort (n = 206), *GSTM1* and *GSTT1* deletions were observed in 49.51% and 25.24% of individuals, respectively. Stratified analysis by region showed that the frequency of the *GSTM1* null genotype was comparable between Cusco and Junín (46.43% vs. 56.06%, *p* = 0.197). Similarly, no statistically significant differences were detected in the frequency of the *GSTT1* null genotype between the two regions (22.14% vs. 31.82%, *p* = 0.139).

Analysis of the combined distribution of GST genotypes revealed that 11.65% of participants carried simultaneous null genotypes for both *GSTM1* and *GSTT1*, whereas 63.11% exhibited at least one null genotype. Regional stratification revealed no statistically significant differences between Cusco and Junín, either for the presence of both null genotypes (9.29% vs. 16.67%, *p* = 0.124) or for the presence of at least one null genotype (59.29% vs. 71.21%, *p* = 0.094).

Comparative analyses were conducted between the Peruvian Andean population included in this study and several reference populations (Table 2), recognizing that methodological differences across studies may limit direct comparability. No statistically significant differences were observed in the frequencies of *GSTM1* and *GSTT1* null genotypes between our Andean cohort and the Peruvian coastal population.

When comparing our cohort with other populations across the American continent, the frequency of the *GSTM1* null genotype was comparable to those reported in Argentina, Bolivia, Brazil, Chile, Venezuela, and northern Mexico; however, it was significantly higher than the frequencies observed in populations from Colombia and central Mexico. Regarding the *GSTT1* null genotype, the frequencies observed in our population were comparable to those reported in Bolivia, Brazil, Colombia, and Venezuela, but were higher than those documented in Argentina, Chile, and Mexico.

When extending the comparison to populations from other continents, the frequency of the *GSTM1* null genotype in our cohort was similar to that reported in populations from Europe, North Africa, North–Central–East Asia, and the Middle East. In contrast, a higher frequency was observed compared with populations from Sub-Saharan Africa, South Asia, and Native American groups. For the *GSTT1* null genotype, our cohort exhibited frequencies comparable to those described in Europe, North–South–Central Asia, and the Middle East, while showing lower frequencies than those reported in Sub-Saharan Africa, North Africa, and East Asia, but higher frequencies relative to Native American populations.

## 3. Discussion

The predominance of the *GSTM1* null genotype over *GSTT1* observed in our cohort mirrors the pattern reported in most global populations, reinforcing the notion that *GSTM1* deletion represents the more frequent loss-of-function variant worldwide (Table 2).

The lack of significant differences in the frequencies of *GSTM1* and *GSTT1* null genotypes between Cusco (southeastern Andes) and Junín (central Andes) indicates a genetically homogeneous distribution of these polymorphisms among the high-Andean populations studied. This finding is consistent with previous evidence of strong genetic continuity across Peruvian high-Andean regions, as demonstrated by both targeted genetic polymorphism analyses [15] and higher-resolution genomic studies [16], reflecting the shared demographic history characteristic of these populations. Moreover, the high proportion of individuals carrying at least one null genotype (63.11%) indicates a substantial population-level prevalence of *GSTM1* and *GSTT1* loss-of-function genetic variants, which may be of particular relevance for interpreting genetic susceptibility patterns in rural settings characterized by frequent environmental and pharmacological exposures, as suggested by previous studies in exposed populations [2,9,17]. In this context, the elevated prevalence of null genotypes in the Andean population underscores the need to generate locally relevant genetic baseline data to support future association studies and inform the development of public health research frameworks, environmental surveillance initiatives, and pharmacogenetic investigations tailored to these communities. Moreover, given the extensive evidence linking *GSTM1* and *GSTT1* null genotypes to increased susceptibility to multiple diseases [8], the prevalence observed in this population provides an important genetic context for future disease-focused studies.

Comparisons with previously studied populations showed that the frequencies of *GSTM1* and *GSTT1* null genotypes in our Andean cohort are similar to those reported in Peruvian coastal populations, suggesting that despite geographical and sociohistorical differences, these polymorphisms do not exhibit marked population structure between these regions. This pattern contrasts with findings for other genetic markers, for which population structure has been observed according to the geographical regions of Peru [18]. These results indicate that studies with broader population coverage are needed to determine whether the uniform distribution of these genotypes reflects intrinsic evolutionary patterns or more recent processes of population admixture.

At the continental level, the frequencies of the *GSTM1* null genotype in our Andean population were comparable to those reported in most South American countries and in northern Mexico. Likewise, *GSTT1* null genotype frequencies were consistent with the South American pattern described in countries such as Bolivia, Brazil, Colombia, and Venezuela. Differences in *GSTM1* and *GSTT1* null genotype frequencies between our Andean cohort and certain populations across the Americas may be attributable to latitudinal variation, as the frequency of the *GSTM1* null genotype increases with absolute latitude, whereas that of the *GSTT1* null genotype decreases [10].

**Table 2:** Comparative distribution of the *GSTT1* and *GSTM1* null genotypes.

Populations	*GSTM1* Null		*GSTT1* Null		References
	n	%	n	%	
Andean Peruvian	206	49.51	206	25.24	Present study
Coastal Peruvian (Lima–Hospital + Community)	100	51.00	-	-	[11]
Coastal Peruvian (Ica and Lima)	131	47.00	-	-	[12]
Coastal Peruvian (Lima–General Population)	81	47.00	-	-	[13]
Coastal Peruvian (Lima and Callao)	377	47.21	377	30.24	[14]
Argentina	609	45.00	609	17.00 *	[19]
Bolivia	297	54.00	297	31.00	[20]
Brazil	594	43.90	594	23.10	[21]
Chile	260	41.80	260	13.20 *	[22]
Colombia	174	35.63 *	174	30.45	[23]
Venezuela	174	45.81	174	19.55	[24]
Mexico North East	211	44.00	211	11.00 *	[25]
Central Mexico	529	33.00 *	529	12.00 *	[26]
European	146,981	51.26	117,789	19.89	[8]
Sub-Saharan African	9867	28.70 *	7011	35.48 *	[8]
North African	3839	53.46	3518	33.35 *	[8]
East Asian	70,088	53.49	57,560	46.63 *	[8]
South Asian	32,557	42.00 *	27,902	25.19	[8]
North Asian	2246	44.15	2144	26.75	[8]
Central Asian	300	52.00	300	32.00	[8]
Native American	1345	35.75 *	945	14.00 *	[8]
Middle Eastern	18,071	48.64	170,339	26.18	[8]

* Significant difference in the frequency of *GSTM1* and *GSTT1* null genotypes compared with the present study (*p* < 0.05).

Comparatively, the frequencies reported in this study also fall within the range documented for populations from Europe, the Middle East, and Asia, but differ from the prevalences reported in Sub-Saharan African and Native American populations. These patterns are consistent with the variability described in global reviews, which report wide ranges of deletion frequencies for both genes according to continental and ethnic origin [8]. Collectively, these findings reinforce the notion that the distribution of *GSTM1* and *GSTT1* null genotypes is not determined solely by national-level geographic criteria, but rather reflects a global pattern shaped by evolutionary history, migration, population admixture, and selective pressures. However, these comparisons should be interpreted with caution, as differences in sample size, inclusion criteria, and genotyping methods across studies may affect frequency estimates. Although our results broadly correspond with some South American and global populations, any interpretation regarding evolutionary or geographic patterns remains preliminary, highlighting the need for larger, standardized studies.

Overall, our findings provide the first evidence on the distribution of *GSTM1* and *GSTT1* null genotypes in Peruvian Andean populations, addressing a critical genetic information gap in historically underrepresented Latin American groups and contributing to precision medicine research [8]. Although this study was limited by a moderate sample size and the inclusion of only two Andean regions, these constraints do not compromise the validity of the initial genetic characterization or the consistency of the observed patterns. Given the cross-sectional design and absence of phenotypic measurements, causal or functional inferences cannot be established. Although *GSTM1* and *GSTT1* deletions are recognized loss-of-function variants, functional or clinical outcomes were not evaluated. These limitations define the scope of the present study and underscore the need for future research in larger and more diverse populations, incorporating genome-wide and gene–environment approaches to better clarify the potential functional and clinical relevance of these polymorphisms and to inform the development of public health research initiatives and precision medicine strategies tailored to high-Andean communities.

## 4. Materials and Methods

### 4.1. Subjects

A cross-sectional study was conducted to determine the prevalence of *GSTM1* and *GSTT1* null genotypes among individuals. Participants were recruited from six localities in the Cusco and Junín regions, comprising a total of 206 individuals selected through regional health centers and local community outreach. Individuals with known chronic liver disease or prior chemotherapy were excluded. Informed consent was obtained from each subject before the study. This study was approved by the Ethics in Research Committee of the Universidad Continental of Peru (N°047-2024-CIEI-UC) and follows the principles of the Declaration of Helsinki. Informed consent was obtained from all the participants.

### 4.2. Sample Collection and DNA Extraction

Peripheral blood samples (3 mL) were collected in ethylenediaminetetraacetic acid tubes and stored at −80 °C until processing. Genomic DNA was extracted using the PureLink Genomic DNA Mini Kit (Invitrogen, Carlsbad, CA, USA), following the manufacturer’s protocols. DNA purity and concentration were evaluated using a NanoDrop Lite spectrophotometer (Thermo Fisher Scientific, Waltham, MA, USA), and DNA integrity was assessed by agarose gel electrophoresis.

### 4.3. Genotyping Analysis

**Figure 1:**
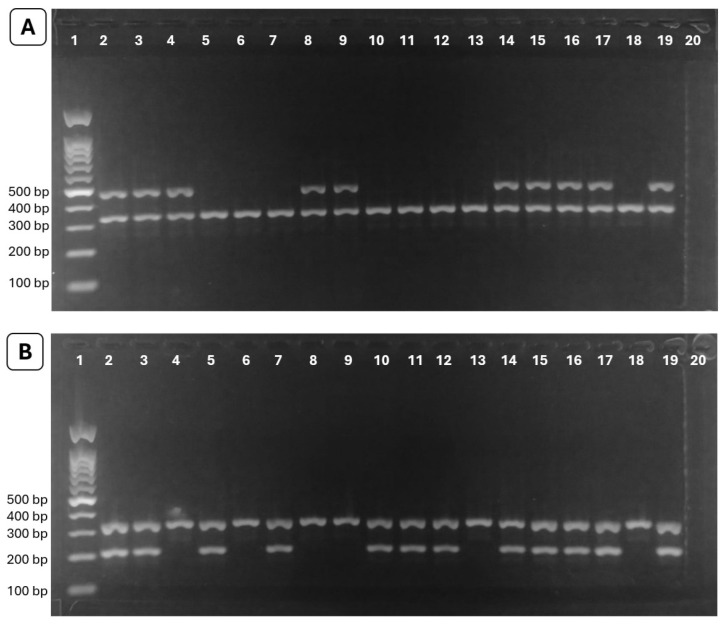
Representative PCR amplification of *GSTT1*, *GSTM1*, and the internal control (human albumin) resolved on 1.5% agarose gels. (**A**) A 480 bp band indicates the non-null *GSTT1* genotype. (**B**) A 215 bp band indicates the non-null *GSTM1* genotype. The albumin band (~300 bp) confirms successful amplification. Lanes 1–18 correspond to representative samples, lane 19 to the positive control, and lane 20 to the no-template control (NTC). Absence of *GSTT1* or *GSTM1* amplification in the presence of the albumin band indicates a null genotype consistent with homozygous deletion.

Genomic DNA regions corresponding to the *GSTM1* and *GSTT1* genes were amplified by PCR using GoTaq^®^ G2 Green MasterMix (Promega, Madison, WI, USA). The following primers were used: 5′-GAACTCCCTGAAAAGCTAAAGC-3′ and 5′-GTTGGGCTCAAATATACGGTGG-3′ for *GSTM1*, and 5′-TTCCTTACTGGTCCTCACATCTC-3′ and 5′-TCACCGGATCATGGCCAGCA-3′ for *GSTT1* [14]. Human albumin was included as an internal amplification control in each reaction using the primers 5′-GCCCTCTGCTAACAAGTCCTAC-3′ and 5′-GCCCTAAAAAGAAAATCGCCAATC-3′ to verify DNA integrity and prevent false-negative results due to PCR failure. All PCR reactions were performed in duplicate in independent experiments conducted at different times to ensure genotyping accuracy, reproducibility, and result consistency. Each 25 µL reaction contained 100 ng of genomic DNA, 0.2 µM of each GST primer pair (*GSTM1* or *GSTT1*), 0.15 µM of each albumin primer, and 12.5 µL of GoTaq^®^ G2 Green MasterMix. The thermal cycling conditions consisted of an initial denaturation at 95 °C for 5 min, followed by 35 cycles of 95 °C for 30 s, 58 °C for 30 s, and 72 °C for 30 s, with a final extension at 72 °C for 4 min. Amplified products were resolved on a 1.5% agarose gel prepared in 1X TAE buffer and stained with GelRed^®^ (Biotium, Fremont, CA, USA). Amplicons of approximately 215 bp for *GSTM1* and 480 bp for *GSTT1* confirmed the presence of the corresponding genes (non-null genotypes), whereas absence of amplification indicated null genotypes consistent with homozygous gene deletions; the internal control generated an amplicon of approximately 300 bp (Figure 1).

### 4.4. Statistical Analysis

Genotype frequencies were expressed as percentages with 95% confidence intervals (CIs), calculated using the Wilson score method. In addition, unadjusted odds ratios (ORs) with 95% CIs were estimated using simple logistic regression models to quantify the association between region (Cusco vs. Junín) and *GSTM1* and *GSTT1* null genotypes. Differences in genotype frequencies between populations were assessed using the chi-square test or, when appropriate, Fisher’s exact test. Given the descriptive and exploratory nature of this molecular epidemiological study, no multivariate modeling was performed. Data analysis was performed using Stata v15 (StataCorp, College Station, TX, USA) considering a statistical significance of *p* < 0.05.

## 5. Conclusions

This study provides the first characterization of *GSTM1* and *GSTT1* null genotype frequencies in Andean populations of Peru, revealing a high prevalence of loss-of-function genetic variants and a homogeneous distribution of these polymorphisms between Cusco and Junín. In historically underrepresented high-altitude populations, these results provide a crucial population-specific genetic baseline related to xenobiotic metabolism. This baseline supports future functional studies, analyses of gene–environment interactions, and association research focused on disease, environmental exposure, or pharmacogenetics aimed at improving the understanding of genetic variability in Andean communities.

## Figures and Tables

**Table 1:** Distribution of *GSTM1* and *GSTT1* null genotypes in the study population.

Genotype	Total n = 206 % (95% CI)	Cusco n = 140 % (95% CI)	Junín n = 66 % (95% CI)	OR (95% CI)	*p*-Value
*GSTM1* null	49.51% (42.76–56.29)	46.43% (38.37–54.67)	56.06% (44.08–67.37)	1.48 (0.82–2.68)	0.197
*GSTT1* null	25.24% (19.80–31.59)	22.14% (16.06–29.71)	31.82% (21.85–43.79)	1.64 (0.84–3.19)	0.139
Both *GSTM1* and *GSTT1* null	11.65% (7.96–16.75)	9.29% (5.51–15.24)	16.67% (9.57–27.43)	1.95 (0.83–4.60)	0.124
At least one null genotype *GSTM1* or *GSTT1*	63.11% (56.33–69.40)	59.29% (51.01–67.07)	71.21% (59.36–80.73)	1.70 (0.90–3.20)	0.094

Genotype frequencies are presented as percentages with 95% confidence intervals (CIs). Odds ratios (ORs), 95% CIs, and *p*-values were estimated using simple logistic regression models comparing Junín to Cusco.

## Data Availability

The original contributions presented in this study are included in the article. Further inquiries can be directed to the corresponding author.
